# Large Animal Models for Foamy Virus Vector Gene Therapy

**DOI:** 10.3390/v4123572

**Published:** 2012-12-07

**Authors:** Grant D. Trobridge, Peter A. Horn, Brian C. Beard, Hans-Peter Kiem

**Affiliations:** 1 Department of Pharmaceutical Sciences, Washington State University, 99164-6534, Pullman, WA, USA; 2 School of Molecular Biosciences, Pullman, WA, USA; 3 Institute for Transfusion Medicine, University Hospital Essen, Virchowstr. 179, 45122 Essen, Germany; E-Mail: Peter.Horn@uk-essen.de (P.A.H.); 4 Fred Hutchinson Cancer Research Center Clinical Research Division, 98109-1024, Seattle, WA, USA; E-Mail: bbeard@fhcrc.org (B.C.B.); 5 University of Washington School of Medicine, Seattle, WA, USA

**Keywords:** foamy virus vectors, gene therapy, hematopoietic stem cells, animal models

## Abstract

Foamy virus (FV) vectors have shown great promise for hematopoietic stem cell (HSC) gene therapy. Their ability to efficiently deliver transgenes to multi-lineage long-term repopulating cells in large animal models suggests they will be effective for several human hematopoietic diseases. Here, we review FV vector studies in large animal models, including the use of FV vectors with the mutant O^6^-methylguanine-DNA methyltransferase, MGMTP140K to increase the number of genetically modified cells after transplantation. In these studies, FV vectors have mediated efficient gene transfer to polyclonal repopulating cells using short *ex vivo* transduction protocols designed to minimize the negative effects of *ex vivo* culture on stem cell engraftment. In this regard, FV vectors appear superior to gammaretroviral vectors, which require longer *ex vivo* culture to effect efficient transduction. FV vectors have also compared favorably with lentiviral vectors when directly compared in the dog model. FV vectors have corrected leukocyte adhesion deficiency and pyruvate kinase deficiency in the dog large animal model. FV vectors also appear safer than gammaretroviral vectors based on a reduced frequency of integrants near promoters and also near proto-oncogenes in canine repopulating cells. Together, these studies suggest that FV vectors should be highly effective for several human hematopoietic diseases, including those that will require relatively high percentages of gene-modified cells to achieve clinical benefit.

## 1. Introduction

Foamy viruses (FVs) have several desirable properties that have led to their development as vectors for hematopoietic stem cell (HSC) gene therapy. These include a large transgene capacity [1], lack of pathogenicity of the parent virus [2] and a broad tropism [3]. Although FV vectors require mitosis for transduction, they are able to form a stable transduction intermediate in serum-starved cells that are later stimulated to divide [4]. This property likely explains why FV vectors have shown great promise for HSC gene therapy, where the target is a quiescent cell that must progress through the cell cycle following infusion in order to self renew and repopulate a recipient. The requirement of FV vectors for mitosis also explains why FV vectors have not been extensively evaluated for gene delivery to post-mitotic tissues, such as muscle. Thus, here we will focus on studies where FV vectors have been evaluated for HSC gene therapy in large animal models using vectors derived from the so-called “human foamy virus”, which is now considered to be a chimpanzee virus isolated from human cells, SFVcpz(hu). This isolate is now called prototypic foamy virus, PFV.

Studies in mouse models paved the way for these large animal studies [5,6,7,8], and mouse models continue to be useful to improve and test novel FV vectors for HSC gene therapy [9,10]. However, there are several limitations of mouse models that necessitate the use of large animals [11]. These limitations include differences between mouse and human stem cell kinetics, the inability to assess long-term repopulation and an inability of mouse models to accurately predict HSC gene transfer efficiency in patients. For example, a human produces a similar number of red blood cells in one day to what a mouse produces over a two-year life span [12]. Thus, large animal studies can provide critical preclinical data to advance HSC gene therapy studies into the clinic. Here, we review progress in the canine and non-human primate models for FV HSC gene therapy.

## 2. Advantages of Large Animal Models for HSC Gene Therapy

There are several advantages of large animal models for HSC gene therapy [11,13]. A critical consideration for efficient transduction with FV vectors is the cell cycle status [4,14], and this is better modeled in large animals. It has been estimated that approximately 75% of mouse HSCs are outside G_0_ [15], and that mouse HSCs divide every 2.5 weeks [16]. In contrast, studies in baboons and macaques suggest that HSCs divide approximately every 36 weeks, more similar to human HSCs, which are estimated to divide every 45 weeks [17,18]. Additionally, in many mouse studies, donor mice are treated with 5-fluorouracil to increase cell cycling in target cells. This approach will not likely be used in a clinical setting, due to a loss of repopulation potential observed when cells are stimulated into cell cycle [19,20,21]. Increasing cycling increases the efficiency of retroviral transduction and can result in mouse models overestimating the efficiency of gene transfer that can be obtained in the clinic.

An important aspect of HSC gene therapy is the use of conditioning regimens to prepare recipients for gene-modified HSCs. Conditioning regimens are designed to enhance the engraftment of gene modified cells and for many hematopoietic diseases, even those where corrected cells have a selective advantage, conditioning of recipients prior to infusion of gene-modified cells will be essential. Extensive experimentation with conditioning regimens has shown that dog models are more predictive than mouse models [22,23]. For example, in immunodeficient mice, even nonmyeloablative conditioning allows robust engraftment of xenogeneic cells.

A critical goal of large animal models for HSC gene therapy is assessing safety. In the French SCID-X1 clinical HSC gene therapy trial using gammaretroviruses, several patients developed leukemia related to vector integration. The term genotoxicity has been used to describe the unwanted side-effect of an integrated vector provirus altering host gene expression. Vector-mediated genotoxicity can occur via several mechanisms, including activation of nearby host genes by enhancers with the provirus (reviewed in [24]). Genotoxicity studies in large animal models are expected to better predict outcomes in patients in part due to the closer stem cell kinetics. Also, integration site data in long-term repopulating cells is not possible in short-lived rodents. Supporting the above, gene therapy studies in large animals have been critical to advance HSC gene therapy and, unlike mouse models, have predicted efficacy in clinical trials for gammaretroviral and lentiviral vectors. 

## 3. Canine Studies of FV HSC Gene Therapy

### 3.1. Efficient Multi-Lineage Gene Transfer Using FV Vectors

The dog model has several advantages, including the fact that dogs can be easily cared for and are less expensive to purchase and maintain than nonhuman primates. The target cell for HSC gene therapy in humans is currently CD34-enriched cells, and both human and dog CD34^+^ cells have similar characteristics *in vitro* and also *in vivo* [25]. The first large animal study evaluating FV vectors was performed in the dog model using an advanced replication-incompetent FV vector [26]. This vector expressed a green fluorescent protein from a phosphoglycerate kinase promoter (PGK) to enable accurate and convenient evaluation of gene marking in myeloid and lymphoid lineages by flow cytometry. In this study, stable multi-lineage marking was observed in two transplanted dogs with approximately 15% of peripheral blood granulocytes and lymphocytes expressing enhanced green fluorescent protein (EGFP) long-term [26]. In this study, preliminary data from *in vitro* colony forming unit (CFU) assays indicated that FV vectors could efficiently transduce canine CD34-enriched cells using a short *ex vivo* culture. The ability of FV vectors to efficiently transduce canine CD34^+^ cells using a short transduction protocol is an important advantage, since it avoids the deleterious effects of *ex vivo* culture on stem cell engraftment [19,20]. Promising results in preliminary CFU assays were borne out *in vivo*. Canine CD34-enriched cells exposed to FV vectors *ex vivo* using an 18-hour transduction protocol resulted in efficient marking and, also, rapid engraftment. Gammaretroviral vectors require a longer *ex vivo* stimulation for efficient transduction of canine CD34^+^ cells [27,28], presumably to allow for stimulation of target cells into the cell cycle. Although FV vectors require mitosis for transduction, a direct comparison of the stability of FV and gammaretroviral vectors in quiescent cells shows that FV vectors are more stable and that FV vectors remain viable in G_0_ cells until these cells are stimulated to divide [4]. Thus, one of the advantages of FV vectors for HSC gene therapy appears to be the stability of a transduction intermediate in quiescent HSCs that are stimulated to divide after infusion [4,29]. This may explain why minimizing *ex vivo* culture by using a short *ex vivo* exposure to vector preparations is sufficient for efficient gene transfer. Another observation from this study was that long-term marking was stable, unlike marking with gammaretroviral vectors, which in previous studies was observed to decline over time, indicating less efficient transduction of cells with long-term repopulating ability [27,28].

FV vectors efficiently deliver transgenes to all blood lineages examined in the above study, including peripheral blood granulocytes, T lymphocytes, monocytes and bone-marrow-derived CD34^+^ cells. Gene expression was also observed in erythrocytes and platelets. Linear ampliﬁcation-mediated-polymerase chain reaction (LAM-PCR) was performed on canine DNA isolated from peripheral blood leukocytes and indicated that all canines were reconstituted with polyclonal populations of hematopoietic repopulating cells. Sequence analysis of individual LAM-PCR products identified individual provirus integration sites, which can be used to identify individual repopulating clones. For two of these clones, quantitative PCR showed that they were present in both highly-purified myeloid and lymphoid peripheral blood cells, strongly suggesting FV-mediated transduction of a multipotent repopulating cell with both lymphoid and myeloid potential. Similar transgene expression levels in primitive bone marrow-derived cells and in mature peripheral blood cells suggest that FV transduction had no deleterious effect on HSC differentiation. This study paved the way for evaluating FV vectors for therapeutic gene transfer, including studies for canine leukocyte adhesion deficiency (CLAD) [30], and also studies exploring FV vectors for pyruvate kinase (PK) deficiency [31] described below (Section 3.3, Section 3.4). The above dogs were used to perform the first comparison of genotoxicity for gammaretroviral, FV and lentiviral vectors in a large animal model, which showed that FV vectors may be safer than gamma or lentiviral vectors [32]. Details of these studies are described below (Section 3.5).

### 3.2. Comparison of FV Vectors to Lentiviral Vectors

**Figure 1:**
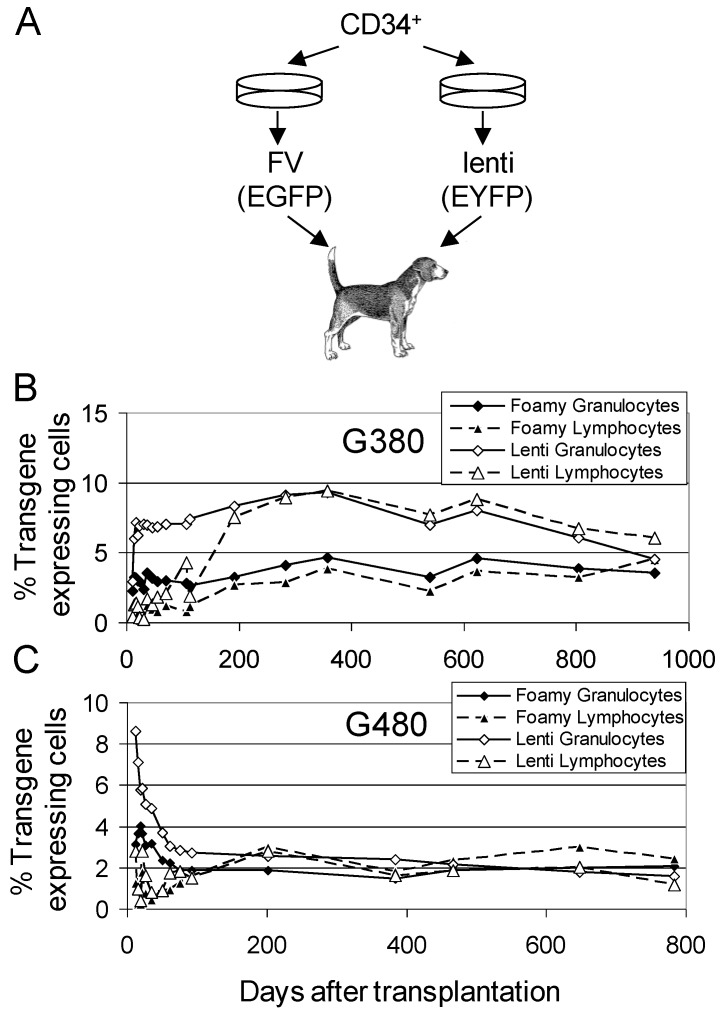
**Competitive repopulation assay of FV and lentiviral vectors in the dog. (a)** The *ex vivo* transduction is divided into two experimental arms with equivalent numbers of CD34^+^ cells. A FV vector expressing EGFP is used for one experimental arm, and a lentiviral vector expressing EYFP is used for the other experimental arm. By directly comparing these vector types in the same dog, inter-animal variability is eliminated, and gene transfer can be evaluated using a small number of animals. The percentage of EGFP and EYFP expressing leukocytes detected by flow cytometry are shown for two dogs G380 (**b**) and G480 (**c**) at different times after transplantation. Panels (**b**) and (**c**) are reproduced from [35].

In the above study, the genetic marking with FV vectors compared favorably to a similar study using lentiviral vectors with a similar short *ex vivo* protocol; however, the lentiviral vectors were used at a much higher multiplicity of infection (MOI). To better compare FV and HIV-based lentiviral vectors, these two vector systems were directly compared in dogs using a competitive repopulation approach [33,34], Figure 1. In this approach, the CD34^+^ target cells are divided into two equal aliquots, and each of these experimental arms is transduced with either a FV or lentiviral vector. Infusion of both experimental arms into the same animal allows a direct comparison of engraftment of gene-modified cells in the same animal, thereby eliminating inter-animal variability. A competitive repopulation experiment performed with an EGFP-expressing FV vector and an enhanced yellow fluorescent protein (EYFP)-expressing HIV-based lentiviral vector in two dogs using a low MOI of 5 for both vector systems resulted in very similar long-term (>700 days) marking [35]. For both vectors, an internal PGK promoter was used to express EGFP or EYFP. Multi-lineage marking was observed in both dogs with both vector types. Long-term marking for both FV and lenti vectors in the two dogs was approximately 5% and 2%, respectively, in peripheral blood lymphocytes and granulocytes. Because each dog received two experimental arms, the total expected marking for the FV and the lentiviral vector experimental arms if just one arm were performed can be estimated approximately at 10% in dog G380 (2 × 5%) and 4% in dog G480 (2 × 2%). Thus, despite the fact that FV vectors require mitosis and lentiviral vectors do not, FV vectors transduce dog long-term repopulating cells at similar frequency to lentiviral vectors. This may be due to the fact that both FV and lentiviral vectors transduce quiescent G_0_ cells that are later stimulated to divide at similar frequency [4]. Also, lentiviral vectors transduce actively dividing cells more efficiently than quiescent G_0_ cells.

### 3.3. FV Correction of Canine Leukocyte Adhesion Deficiency (CLAD)

The above studies set the stage for using FV vectors to cure a disease via HSC gene therapy in a large animal model. Previous studies had shown that CLAD, a severe genetic immune deficiency that results in recurrent life-threatening bacterial infections, could be corrected using HSC gene therapy with gammaretroviral vectors [36]. CLAD is an autosomal recessive diseases that results from a single point mutation in the CD18 leukocyte integrin subunit, which prevents surface expression of the CD11-CD18 leukocyte integrin complex. The disease models leukocyte adhesion deficiency-1 (LAD1) in humans in which patients suffer recurrent, life threatening bacterial infections due to an inability of their neutrophils to adhere to blood vessel walls and extravasate into tissues at the sites of infection. A FV vector expressing canine CD18 from an internal murine stem cell virus promoter was used to transduce CLAD dog CD34^+^ bone marrow cells. Five dogs were treated with FV-transduced autologous CD34 cells, but one dog died from a transplantation-related intussusception, unrelated to FV vector use. Long-term gene transfer to myeloid and lymphoid cells was achieved in all four surviving dogs with no evidence for transgene silencing. These four dogs all had reversal of the CLAD phenotype that persisted long-term (>2 years). In this study, 5–10% of peripheral blood leukocytes were transduced and 3–7% of bone marrow cells were transduced. The use of the strong murine stem cell virus promoter in this study was of concern due to the possibility it may activate neighboring host genes. A later study was also performed using the housekeeping PGK promoter, which is expected to have a lower propensity to activate nearby host genes [37]. However, this study failed to show efficacy. Two of the dogs never achieved more than 1% CD18^+^ cells. Higher gene marking rates were measured by qPCR, and the authors suggested the low transgene expression *in vivo* might be due to inadequate expression for flow cytometry analysis or silencing of the CD18 transgene. It is unclear why the PGK promoter failed in this study, but it highlights the fact that FV vector design and vector components can be critically important to the outcome of preclinical studies and likely, also, clinical trials.

### 3.4. Correction of Canine Pyruvate Kinase (PK) Deficiency

For many immune deficiencies, relatively low gene transfer efficiency is all that is required to correct the disease phenotype. In the above CLAD study, gene marking of less than 10% was able to result in a corrected phenotype. For HSC gene therapy clinical studies of severe combined immunodeficiency SCID-X1, even lower gene marking is sufficient for therapeutic efficacy due to the strong selective advantage mediated by the corrective transgene (γC) [38]. However, for many other hematopoietic diseases, such as thalassemias, more efficient gene transfer will be required. For example, it has been estimated that at least 20% of erythroid precursors would need to be gene-modified to provide significant therapeutic benefit to patients with severe beta thalassemia [39].

PK deficiency in Basenji dogs is associated with severe, chronic hemolytic anemia [40,41]. PK-deficient dogs have residual erythrocyte PK activity mediated by expression of the M2-type PK isoenzyme. The M2 isoenzyme is normally present in all tissues during fetal life and remains the major isoenzyme in erythroid precursors. PK deficient dogs lack the normal R-type isoenzyme, which begins to appear in normal erythrocytes as erythroid maturation proceeds. The expression of the M2-type isoenzyme is thought to be a compensatory change for R-type PK deficiency, but it does not prevent hemolysis *in vivo*. PK deficient dogs have shortened lifespans due to progressive myelofibrosis, osteosclerosis and liver cirrhosis. Allogeneic transplantation studies in the dog suggest that curing PK deficiency will require relatively high, approximately 20%, marking in long-term repopulating cells [40,42,43]. As such, the Basenji dog is a particularly good model for several erythroid diseases, since high levels of marking will likely be required to observe clinical improvement, and unlike severe combined immunodeficient (SCID) models, gene-modified cells should not have a significant selective advantage.

We used the PK-deficient dog model to explore the use of FV vectors that allow for *in vivo* selection to treat hematopoietic diseases, such as PK deficiency and severe thalassemia, that will require relatively high levels of marking [31]. A FV vector containing a P140K O^6^-methylguanine-DNA methyltransferase (MGMTP140K) resistance gene, in addition to the therapeutic canine PK-R transgene, was constructed in order to increase the percentage of cells after transplantation [31]. MGMTP140K confers resistance to methylating agents, such as temozolomide, as well as to nitrosoureas, such as BCNU, and allows efficient selection of long-term repopulating HSCs [44]. The MGMT protein is expressed in normal tissues, so in order to enhance selection, O^6^-benzylguanine (O6BG) is used to inactivate endogenous MGMT. Mutant MGMT genes that confer resistance to O6BG are delivered to HSCs so that alkylating agents, such as BCNU, or temozolomide, used in conjunction with O6BG, allow for efficient selection of transduced HSCs. A FV vector with MGMTP140K was designed to allow for *in vivo* selection post-transplantation to increase the percentage of cells into a therapeutic range. This tri-cistronic FV vector with canine PK-R and MGMTP140K also expressed EGFP to allow for accurate monitoring of gene marking and selection *in vivo*.

A PK-deficient dog was treated with this tri-cistronic FV vector at an MOI of approximately 10, resulting in marking that initially stabilized at approximately 3.5% in peripheral blood granulocytes and 0.4% peripheral blood lymphocytes early (100 days) after transplantation. Three treatments of O6BG and BCNU (O6BG 5 mg/kg and BCNU 0.2, 0.3, and 0.4 mg/kg) were administered to select for MGMTP140K-expressing cells that also express the therapeutic PK transgene. Following these three treatments, marking increased to approximately 33% in granulocytes and 5.5% in lymphocytes. Prior to HSC gene therapy and prior to the third O6BG and BCNU treatment, the dog had required whole blood cell transfusions to treat anemia, as indicated by a drop in the hematocrit to below 20%. However, after the third treatment, the hematocrit stabilized at approximately 25%, resulting in the animal becoming transfusion-independent. There was also an associated decrease in nucleated red blood cells consistent with correction of the PK deficient phenotype. Serum levels of lactate dehydrogenase were normal, indicating a reduction of red blood cell lysis, further confirming therapeutic benefit. In depth gene-modified clonal analysis is ongoing to assess genotoxicity, but the stability of gene marking and absence of clinical indicators of transformation suggest a relatively safe approach. Thus, after FV vector-mediated *in vivo* selection, the PK deficiency was cured. This study suggests that FV vectors that also express MGMTP140K may be useful not only for PK deficiency, but for other severe erythroid diseases, such as hemoglobinopathies, that require a high percentage of corrected cells to attain clinical benefit.

### 3.5. Evaluation of FV Genotoxicity in the Dog Large Animal Model

FV vectors appear to be relatively safe based on *in vitro* studies showing they integrate near proto-oncogenes less frequently than gammaretroviruses and lentiviruses [45] and, also, the fact that they are less likely to transactivate nearby genes than gammaretroviruses or lentiviruses [46]. However, *in vitro* studies are limited in that the effects of genotoxicity on repopulating hematopoietic cells cannot be assessed. By evaluating FV vector integration sites in long-term repopulating cells in large animal models and monitoring potential clonal expansion or progression to leukemia, we can better understand the safety of vectors proposed for clinical trials. A comparison of FV, lentiviral and gammaretroviral vector insertion sites in dog long-term repopulating cells showed that FV vectors were found less frequently within proto-oncogenes than either lentiviral vectors or gammaretroviruses [32]. In this study, all dogs were repopulated with multiple clones of retroviral gene-modified cells with no evidence of progression to monoclonality. Gammaretroviruses were frequently found very close (<2.5 kbp) to the start site of genes. This study suggested that FV vectors may have a lower genotoxic risk than gammaretroviral vectors. 

It is important to note that this study was performed in a genetic background in which there is no expected exogenous selection pressure for gene-modified repopulating cells. To best understand genotoxicity in any given specific disease setting, the relative genotoxicity of different vector types and vector designs should ideally be tested in animal models that recapitulate the disease phenotype. For example, the relative genotoxicity of FV and gammaretroviral vectors for SCID-X1 gene therapy would be best assessed in SCID-X1 animal models where the therapeutic transgene (γC) is known to provide a selective advantage to gene-modified cells. In a SCID-X1 background, any potential genotoxic effects specific to the SCID-X1 disease setting, including γC-mediated expansion of gene-modified cells, can then be modeled. It is interesting to note that in the SCID-adenosine deaminase (ADA) deficiency background to date, no severe adverse events related to insertional mutagenesis have occurred, although there have been reports of integrants near proto-oncogenes in all three trials.

An analysis of FV integration sites was performed in the CLAD study discussed above. In this study, there were no adverse events, and dogs contained a polyclonal population of gene-modified repopulating cells [30]. The frequency of FV integrants within or near oncogenes was compared to a previous study where a gammaretroviral vector was used to treat CLAD dogs. FV vector proviruses were observed within or near proto-oncogenes less frequently than gammaretrovirus vector proviruses in the CLAD disease setting. An analysis of over-represented gene classes near FV vector integrants suggested no clear *in vivo* selection for clones in which the provirus had activated nearby genes. A long-term follow up study that tracked 11 specific FV integrants showed continued polyclonal repopulation with no evidence for clonal expansion [47]. 

In summary, *in vivo* genotoxicity studies show that transduction of HSCs with FV vectors can lead to long-term polyclonal expansion of repopulating cells with as yet no adverse events. While FV vectors do integrate within and near proto-oncogenes in repopulating cells, they do so less frequently than gammaretroviral vectors. Additionally, there has been no evidence for clonal expansion. Long-term follow up of these animals and additional studies in other disease-specific models, such as SCID-X1, will be invaluable to assess the safety of FV vectors proposed for clinical studies.

## 4. Nonhuman Primate Studies of FV Vector HSC Gene Therapy

### 4.1. Challenges Using Nonhuman Primate Models for FV HSC Gene Therapy

**Figure 2:**
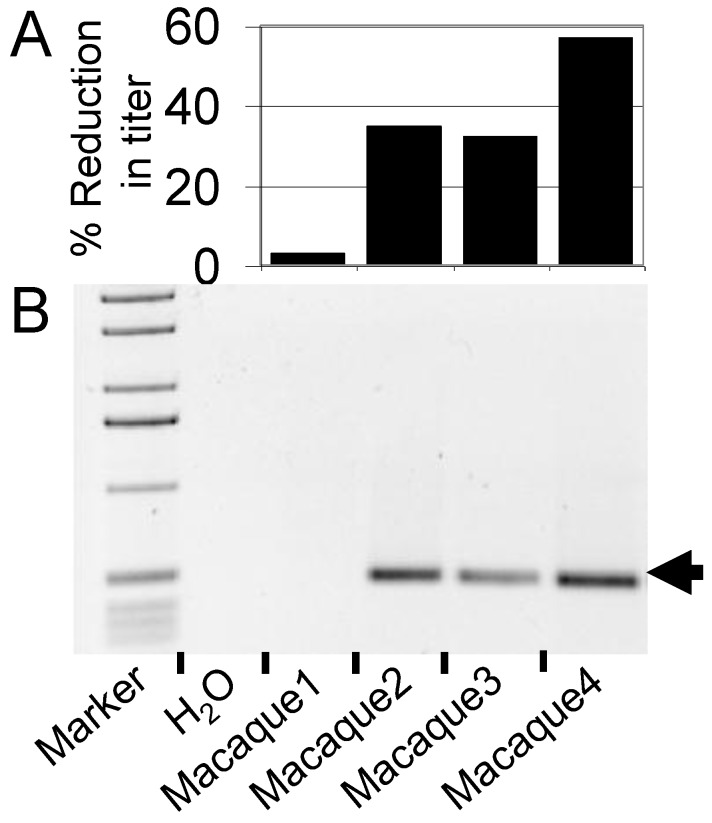
**Identification of suitable macaques for transplantation (a)** Analysis of serum neutralization. Heat-inactivated and non-heat-inactivated serum from four macaque transplant candidates was incubated with 5 × 10^4^ transducing units of an EGFP foamy vector preparation and titered on HT1080 fibrosarcoma cells. The percent reduction in titer of vector incubated with non-heat-inactivated serum relative to vector incubated with heat-inactivated serum is shown. **(b)** Nested PCR screen of macaque peripheral blood for foamy pol. 200 ng of peripheral blood DNA from the four macaque transplant candidates was used to amplify foamy *pol* in a nested PCR reaction. The 465 bp product indicated by an arrow demonstrates the presence of foamy proviral/genomic DNA.

Nonhuman primates are our closest relatives and have some advantages over canine models for HSC gene therapy, including the ability to study diseases such as AIDS [48], which cannot be modeled in dogs. However, wild-type simian foamy virus (SFV) infection is endemic in captive primate species, which complicates the use of nonhuman primates for preclinical FV HSC gene therapy studies. The presence of wild-type SFV in these animals poses a risk of recombination between wild-type SFV and FV vectors. This also means that infected primates may have circulating serum antibodies to SFV. This is a concern, since these antibodies may cross-react with FV vector proteins that may be present on infused cells after *ex vivo* exposure to concentrated FV vector preparations. It is unclear what effect this would have on the efficiency of engraftment of gene-modified cells, but clearly SFV-negative animals are desirable to avoid these potential complications. In our (GDT and HPK) studies with pigtailed macaques (*Macaca nemestrina*), we have developed a simple, but powerful, serum neutralization method to evaluate the relative ability of serum from different macaques to inactivate FV vectors *in vitro*. In this approach, serum from different macaques is pre-incubated with FV EGFP vector preparations for 30 minutes. The serum-exposed vector is then added to cells, and the remaining viable FV vectors transduce these cells and can be detected by EGFP expression. Using this method, we have observed significant differences in serum neutralization between macaques, Figure 2A. We have also employed a previously described PCR screen for FV *pol* DNA [49] in peripheral blood to identify SFV-infected animals, Figure 2B. We have thus chosen monkeys for transplantation that have an undetectable level of *pol* DNA in peripheral blood by PCR and also have a low level of neutralization of FV vectors using this serum screen. While we cannot say with certainty that these monkeys are SFV-negative, we believe this screening approach is a useful method to identify monkeys suitable for transplantation. Both techniques are straightforward, can be rapidly performed and do not require any specialized reagents other than FV vector preparations.

### 4.2. FV Gene Therapy in the Pigtailed Macaque

**Figure 3:**
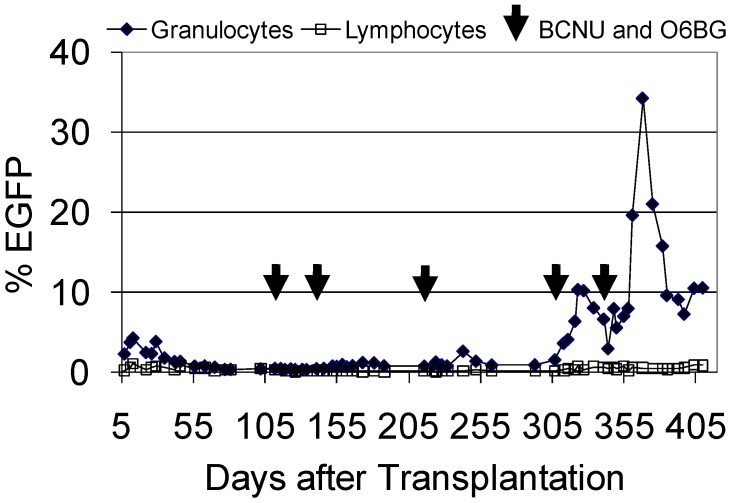
**FV vector marking and *in vivo* selection with MGMTP140K in a pigtailed macaque.** A pigtailed macaque was conditioned with 800 cGy total body irradiation, and 1.7 × 10^7^ CD34-enriched cells exposed to a FV vector at an MOI of 0.2 were infused. The FV vector expresses EGFP and MGMTP140K to allow for convenient tracking and *in vivo* selection. The percentage of EGFP-expressing granulocytes and lymphocytes in peripheral blood is shown.

We performed a transplant in a pigtailed macaque screened using these procedures with a FV vector that expressed MGMTP140K and EGFP (Figure 3). In this experiment, the marking was relatively low, but after a series of treatments with O6BG and BCNU, we were able to increase the percentage of EGFP-expressing granulocytes transiently to over 30%. Unfortunately, this animal died due to transplant-related causes, and we were not able to evaluate marking long-term. However, we did observe stabilization of marking in granulocytes at above 10% (Figure 3). In this monkey, we dose-escalated BCNU concentrations in order to establish dosing, thus we required several treatments to observe efficient selection *in vivo*. We have since established our dosing protocol for O6BG and BCNU in the macaque model, and in future monkeys, we should be able to establish efficient selection with fewer treatments as we have done for lentiviral vectors [50,51] and for FV vectors in the dog model. Clearly, additional FV studies are needed in the macaque model, but this experiment suggests that pigtailed macaques should be an excellent model to evaluate FV vectors and may be particularly useful for AIDS gene therapy studies [10,50].

### 4.3. Progress in the Marmoset Model

The common marmoset *Callithrix jacchus* serves as a very useful nonhuman primate model, because of its small size, unproblematic breeding and long life span [52]. It is thus a relatively inexpensive nonhuman primate model. Recently, marmosets have received increased interest, since they are the only nonhuman primates for which transgenic animals with germline transmission have been generated [53]. Marmosets are commonly infected with FVs, however, since only little data existed on the transduction efficiency of FV vectors for the transduction of marmoset stem cells, we (PAH) performed a direct comparison of marmoset CD34^+^ hematopoietic progenitor cell transduction efficiency using identically designed gammaretroviral, lentiviral and FV vector constructs expressing EGFP from the spleen focus forming virus (SFFV) promoter. FV vectors were pseudotyped with a modified prototype foamy virus (PFV) envelope [54] or with the G-protein of vesicular stomatitis virus (VSV-G), while the gammaretroviral and lentiviral vectors were pseudotyped with VSV-G. We transduced previously cryopreserved CD34-enriched cells from the bone marrow of a common marmoset either after a two day prestimulation in the presence of interleukin-6 (IL-6), Fms-related tyrosine kinase 3 ligand (FLT3L), stem cell factor (SCF) and thrombopoietin (TPO) at a concentration of 100 ng/mL each or after overnight incubation with 100 ng/mL SCF only. Equal numbers of cells were exposed for 16 hours on fibronectin fragment (CH-296, also called RetroNectin)-coated dishes. CH-296 enhances FV transduction of CD34^+^ cells and also inhibits apoptosis during *ex vivo* culture [7,55,56]. The read-out was based on fluorescence microscopy of colonies plated in methyl cellulose, as well as flow cytometry. FV vectors with the FV envelope was the most efficient gene transfer tool for marmoset hematopoietic CD34^+^ cells with stable transduction rates of over 80% as assessed by flow cytometry at both three or eight days after vector exposure and, on average, 88% transduction efficiency into colony forming unit cells (CFU-C). Transduction of CFU-C with the other vectors was always below 60%. In conclusion, we achieved highly efficient gene transfer into common marmoset hematopoietic CD34^+^ cells using FV vectors. These results suggested that FV vectors may be highly feasible vectors for stem cell gene transfer in the marmoset and, thus, set the stage for a more detailed analysis of this vector system in transplantation studies also in this nonhuman primate model [57].

Subsequently, three marmosets were successfully transplanted with autologous FV transduced HSCs. Animals were treated with PEGylated G-CSF five days before bone marrow was collected from the femur of anesthetized marmosets. After density centrifugation, CD34^+^ cells were enriched by magnetic separation using the recently cloned monoclonal mouse anti-marmoset CD34-antibody MA24. The FV vector construct (MH71.MGMT) used expressed EGFP and MGMTP140K, separated by an internal ribosome entry site. The genes were expressed from the SFFV promoter. Vectors were pseudotyped with a modified prototype foamy virus envelope. CD34-enriched cells were transduced at an MOI of approximately 10 in the presence of IL-6, FLT3L, SCF and TPO overnight.

All three animals were transplanted with approximately 4 × 10^5^ CD34^+^ cells per kg body weight after administration of a mild nonmyeloablative conditioning of 1–2 mg/kg busulfan 24 hours prior to transplantation. Hematopoietic recovery was without any complications. Within less than three weeks after transplantation, EGFP positive granulocytes were detectable by flow cytometry. EGFP was detectable for at least one year in all animals. However, marking soon had dropped to levels below the detection limit for flow cytometry, and EGFP could only be detected by PCR at time points later than one month after transplantation. Most likely, this was due to the lack of sufficient conditioning to allow for high level engraftment of transplanted cells. Still, the detection of low level marking at time points later than one year demonstrates transduction of long-term repopulating stem cells by FV vectors also in this non-human primate model.

## 5. Conclusions

FV vectors are able to efficiently deliver genes to large animal HSCs, resulting in long-term multi-lineage repopulation, the most stringent criteria for HSC gene transfer, short of clinical trials. Large animal studies in conjunction with studies showing efficient transduction of human HSCs in immunodeficient mouse models have set the stage for clinical trials with FV vectors. FV vectors have significant advantages over gammaretroviral vectors and also compare favorably with lentiviral vectors based on competitive repopulation experiments in the dog model. Ongoing and future studies in the canine and nonhuman primate models will be important to address the efficacy and genotoxicity of FV vectors in disease-specific settings. Large animal models will also be useful to rigorously test improved vector designs, such as the incorporation of insulator elements and, also, lineage-specific promoters to restrict gene expression to the cellular target of interest.
